# Optimized procurement of cancer-reactive T-cell receptors from clinical samples

**DOI:** 10.1093/immadv/ltag009

**Published:** 2026-06-22

**Authors:** Marine E Caillaud, Cristina Rius, Théo Morin, Li Rong Tan, Hannah L Thomas, Amber Rogers, Joshua Hick, Morten Nielsen, Md Samiul Hasan, Inge Marie Svane, Barbara Szomolay, Andrew K Sewell, Garry Dolton

**Affiliations:** Division of Infection and Immunity, Cardiff University School of Medicine, Cardiff, Wales, United Kingdom; Division of Infection and Immunity, Cardiff University School of Medicine, Cardiff, Wales, United Kingdom; Division of Infection and Immunity, Cardiff University School of Medicine, Cardiff, Wales, United Kingdom; Division of Infection and Immunity, Cardiff University School of Medicine, Cardiff, Wales, United Kingdom; Division of Infection and Immunity, Cardiff University School of Medicine, Cardiff, Wales, United Kingdom; Division of Infection and Immunity, Cardiff University School of Medicine, Cardiff, Wales, United Kingdom; Division of Infection and Immunity, Cardiff University School of Medicine, Cardiff, Wales, United Kingdom; Centre for Cancer Immune Therapy, Herlev Hospital, Copenhagen University, Copenhagen, Denmark; Division of Infection and Immunity, Cardiff University School of Medicine, Cardiff, Wales, United Kingdom; Centre for Cancer Immune Therapy, Herlev Hospital, Copenhagen University, Copenhagen, Denmark; Division of Infection and Immunity, Cardiff University School of Medicine, Cardiff, Wales, United Kingdom; Systems Immunity Research Institute, Cardiff University Cardiff, Wales, United Kingdom; Division of Infection and Immunity, Cardiff University School of Medicine, Cardiff, Wales, United Kingdom; Systems Immunity Research Institute, Cardiff University Cardiff, Wales, United Kingdom; Division of Infection and Immunity, Joint Research Center for Human Retrovirus Infection, Kumamoto University, Kumamoto 8600811, Japan; Division of Infection and Immunity, Cardiff University School of Medicine, Cardiff, Wales, United Kingdom

**Keywords:** cancer, immunotherapy, T-cell receptor sequencing, flow cytometry, viable T-cells

## Abstract

T-cell receptors can sense cancer-associated changes in cellular proteins, lipids, metabolites, and stress pathways, enabling immune-mediated tumour elimination in some patients. These receptors underpin T-cell receptor-based immunotherapies, yet their isolation from clinical samples remains challenging due to labour-intensive workflows, dependence on prior antigen knowledge, and loss of cell viability. We developed an optimized flow cytometry-based assay, termed the T107 assay, to isolate viable cancer-reactive T-cells directly from clinical samples. Following short-term exposure to cancer cells, responding T-cells are identified by simultaneous surface detection of tumour necrosis factor and the degranulation marker CD107a, enabling recovery of live antigen-reactive cells for downstream analysis. The T107 assay enabled rapid identification of cancer-reactive T-cells from tumour-infiltrating lymphocyte products derived from melanoma and sarcoma patients. The assay supported phenotypic and functional characterization of αβ and γδ T-cell receptor populations, including CD4^+^ and CD8^+^ subsets, responding to both patient-specific and shared cancer antigens. Isolation of viable responding cells enabled high-resolution receptor sequencing, generation of functional T-cell clones, determination of antigen specificity and major histocompatibility complex restriction or independence. Paired receptors were engineered into primary T-cells to generate T-cell receptor-engineered products capable of recognizing multiple cancer types. The T107 assay provides a rapid and robust method for antigen-agnostic procurement of viable cancer-reactive T-cells and their receptors from clinical material. This approach removes key bottlenecks in T-cell receptor discovery and is expected to accelerate development of T-cell receptor-based immunotherapies across a broad range of cancers.

## Graphical Abstract

**Graphical Abstract:**
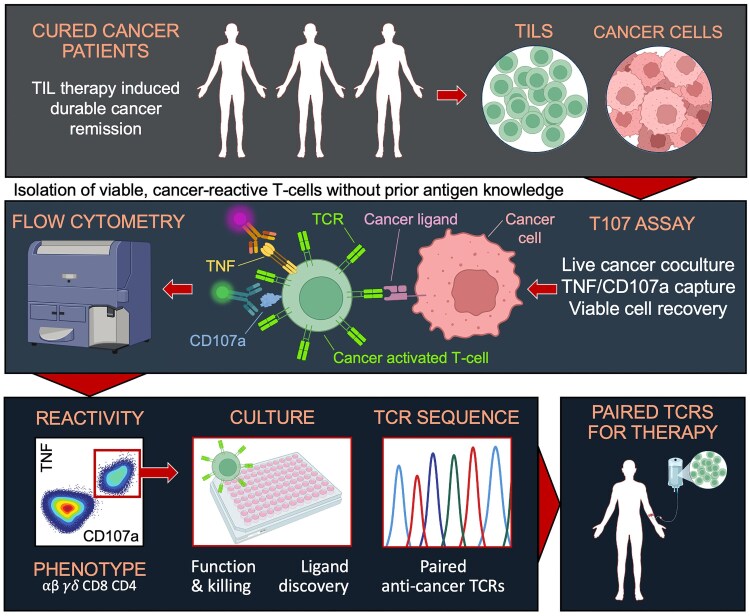

## Introduction

Much recent research has focussed on adoptive cell therapies (ACT) using natural or genetically modified T-cells for treatment of end-stage cancer. Most interest has focused on CD8 ‘killer’ T-cells which have the dual ability to sense the altered proteome in cancerous cells and then directly destroy them using a variety of cytotoxic mechanisms. Conventionally, T-cells recognize processed protein antigens in the form of short peptides bound to HLA molecules at the cell surface [1]. However, it is now realized that the T-cell receptor (TCR) is a far more versatile tool and can recognize ligands including processed lipids and metabolites and unprocessed cell surface stress proteins [2]. This breadth of ligand recognition greatly expands the range of molecular changes associated with malignant transformation that can be surveyed by T-cells. The recognition of cancer via unconventional ligands, such as MHC-related protein 1 (MR1) [3, 4], is attractive as it allows any given treatment to be applicable to all individuals and bypasses the major limitation of HLA restriction when using conventional TCRs. The ability of TCRs to discriminate between healthy cells and cancer cells and orchestrate destruction of the latter has therefore become a major focus of current immunotherapies. Adoptive transfer of tumour-infiltrating lymphocytes (TILs) has induced complete remission in some metastatic melanoma patients and is now being trialled in other cancers with some success [5, 6]. TIL therapy involves tumour excision, isolation and rapid expansion of TILs *ex vivo* prior to reinfusion of the T-cell product back to the patient. Even 20 years ago, objective response rates exceeding 50% and complete durable response rates of >20% were observed in TIL-ACT therapy for metastatic melanoma [7]. Unfortunately, success rates have not improved substantially since then and there is an urgent need to understand why, and how, TIL-ACT is only successful sometimes so that outcomes can be improved. One problem with using autologous TILs for treatment is that only low numbers of, and in some cases none of, the TILs react to cancer cells [8]. This drawback might be overcome by screening and selecting for the TILs that respond to tumour, but this process considerably extends the time and labour required to generate a clinical infusion product. Another popular approach to increase the cancer-reactivity of T-cell infusion product is to genetically engineer the T-cells to equip them all with a cancer-reactive receptor. The most successful example of this approach involves transfer of T-cells expressing a chimeric antigen receptor (CAR) that recognizes CD19 on the surface of B-cells and B-cell tumours [9, 10]. Indeed, the US FDA has approved several CD19-directed CAR-T therapies, including Tisagenlecleucel (Kymriah), Axicabtagene ciloleucel (Yescarta), Brexucabtagene autoleucel (Tecartus), Lisocabtagene maraleucel (Breyanzi), and Obecabtagene autoleucel (Aucatzyl), and has also approved the BCMA-targeted CAR-T therapies Idecabtagene vicleucel (Abecma) and Ciltacabtagene autoleucel (Carvykti) [11]. The B-cell aplasia associated with B-cell antigen-directed therapies is manageable, but loss of healthy T-cells or myeloid cells cannot be tolerated thereby limiting rollout of CAR-T therapy to other hematologic ‘liquid’ cancers. A new approach targeting of the molecular differences between the TCR β-chains encoded by the *TRBC1* and *TRBC2* genes to allow specific targeting of T-cell tumours while sparing approximately half the healthy T-cells may provide a route for treating aggressive T-cell tumours [12]. However, there is a paucity of CAR targets for myeloid and solid tumours and replication of the unprecedented clinical outcomes in the treatment of some haematologic cancers by CAR-T therapy seems unlikely for the vast majority of cancer in the absence of a major breakthrough. In the meantime, there has been an increasing focus on engineering T-cells with enhanced, or natural, cancer-specific TCR genes prior to ACT, so called TCR-T cell therapy. Coupled with the success of TIL therapy and T-cell checkpoint blockade, current evidence suggests that TCR-T therapy is more suitable for treatment of solid tumours than CAR-T therapy [13, 14].

Numerous studies have linked tumour-infiltrating T-cells to progression-free survival in many cancer types [15–20]. This assertion has been further supported by the durable responses induced in some patients by treatment with the ‘T-cell growth factor’ IL2 [21, 22] or TIL therapy as discussed above. More recently, the advent of T-cell checkpoint blockade has allowed T-cell-mediated regression in a wide range of cancers [23–29] to put the idea that natural T-cells can clear cancer beyond any doubt. This success has focussed attention on what antigens T-cells target during successful therapy and cultivated a voracious appetite for TCRs that target new tumour-associated antigens for use in TCR-T therapies. Unfortunately, generation of optimal cancer-specific T-cells and TCRs remains a major bottleneck to TCR-T therapy. Traditionally, antigen-specific TCRs have been generated by laborious cell culture and cloning using known, or presumed, peptide targets. We recently advanced this technique by using T-cell libraries to allow simple parallel generation of multiple peptide-specific human T-cell clones [30]. While a welcome improvement on previous cell cloning techniques, this approach still requires ∼6 weeks to generate a fully validated T-cell clone for any given antigen and also necessitates prior knowledge of the antigenic peptide. Another common way of identifying and procuring cancer-specific TCRs is by using peptide-HLA multimers to pull out T-cells from peripheral blood mononuclear cells (PBMCs) by flow cytometry [31, 32]. Several recent improvements to this technique enhance staining of cancer-specific T-cells [33, 34] or enable parallel detection of antigen-specific T-cells by combinatorial encoding of peptide-HLA multimers [35] but these techniques require knowledge of the TCR ligand and an ability to manufacture it in soluble form to build the multimeric reagents required to overcome the intrinsic weak affinity of natural TCR-pHLA interactions [31, 32]. Additional technologies have been applied to allow unbiased identification of tumour-reactive TCRs within oligoclonal T-cell populations by TCR gene capture [36] but have not been widely adopted. Here we revisit the procurement of cancer-specific TCRs from clinical samples and provide an updated methodology that we have successfully used to generate cancer-specific CD4, CD8 and γδ T-cells and TCRs without *a priori* knowledge of what antigens(s) these cells respond to.

## Materials and methods

### Patient and healthy donor samples

TIL and/or autologous cancer cell lines were obtained from patients MM909.06 (melanoma line), MM909.11 (TIL and melanoma line), MM909.14 (melanoma line), MM909.15 (TIL and melanoma line), MM909.22 (melanoma line), MM909.24 (TIL and melanoma line), MM909.46 (TIL and melanoma line) and SAR26 (TIL and sarcoma line) enrolled at the Center for Cancer Immune Therapy in Denmark (CCIT-DK) [5, 37]. Details for patients with both TILs and autologous cancer cell lines are provided in Table 1 [5, 37, 38]. TILs from melanoma patients MM909.11, MM909.15, MM909.24 and MM909.46, and sarcoma patient SAR26, were generated from resected tumour lesions using the ‘young TIL’ protocol consisting of an initial pre-rapid expansion phase [39] followed by a rapid expansion procedure [40]. Healthy donor blood samples were sourced from the Welsh Blood Service (Velindre NHS Trust, Wales, UK), with informed consent provided as part of the standard donation procedure. All samples were used under local ethical approval granted by the School of Medicine Research Ethics Committee (reference 18/56). Healthy donor blood was provided as EDTA-treated buffy coats and confirmed seronegative for HIV-1, HBV, and HCV. Blood and derived cells were handled in accordance with Cardiff University guidelines and in alignment with the United Kingdom Human Tissue Act 2004. PBMCs were separated from whole blood as previously described [41].

**Table 1:** Patient information and clinical responses for this study.

Patient ID	Age (sex)	Disease	Outcome	Treatment	Number of TILs infused	Reference
MM909.24	56 (M)	Malignant melanoma	CR	Young TIL	8.6 ×10^10^	[5]
SAR26	89 (F)	Myofibroblastic sarcoma	—	Not treated	—	[37]
MM909.15	48 (F)	Malignant melanoma	CR	Young TIL	11 ×10^10^	[5]
MM909.46	50 (F)	Malignant melanoma	SD	Young TIL	7.5 ×10^10^	[5]
MM909.11	42 (M)	Malignant melanoma	CR	Young TIL	7.5 ×10^10^	[38]

Clinical and demographic information for patients included in this study, including disease indication and clinical response status, defined as complete response (CR) or stable disease (SD).

### T-cell culture

T-cell clones, T-cell lines and TILs were cultured in T-cell media (RPMI-1640 supplemented with 10% heat-inactivated FBS, 100 U/ml penicillin, 100 μg/ml streptomycin, 2 mM L-glutamine; 25 ng/ml interleukin IL-15 (Miltenyi Biotech, Bergisch Gladbach, Germany), 200 IU/ml IL-2 (Proleukin®; Prometheus, San Diego, CA), 1X non-essential amino acids solution, 1 mM sodium pyruvate and 10 mM HEPES buffer (all from Merck KGaA, Darmstadt, Germany, unless stated otherwise) at 37°C with 5% CO_2_. Cells were cultured in 24-well plates at a density of 3–4 × 10^6^ cells per well. Culture medium was replaced by 50% three times per week. Every 2–4 weeks, T-cells were expanded by seeding 0.2 × 10^6^ T-cells per well of a 24-well plate with 4 × 10^6^ irradiated (3100 cGy) PBMCs from three allogeneic donors and 1 µg/ml L-phytohemagglutinin (Remel, Thermo Fisher Scientific) in 2 ml of T-cell medium supplemented with 20 IU/ml IL-2. After 7 days, cultures were transitioned to T-cell medium containing 200 IU/ml IL-2. T-cells were used for functional assays from day 14 post-expansion onwards. Because TILs comprise a mixed population of T-cells, their composition and reactivity can change across expansion passages; passages 2–4 were used in this study.

### Cancer cell lines

Cell lines were regularly tested for mycoplasma using a MycoAlert® mycoplasma detection kit according to the manufacturer’s instructions (Lonza, Basel, Switzerland) and cultured at 37°C with 5% CO_2_ in R10, D10 or D10-F12 (RMP1-1640, DMEM or DMEM-F12 with 10% heat-inactivated FBS, 100 U/ml penicillin, 100 μg/ml streptomycin and 2 mM L-glutamine). HLA genotypes of the cell lines are detailed in Table 2. Adherent cell lines were passaged when 50–80% confluent by detachment from tissue culture plates or flasks with D-PBS and 2 mM EDTA, then splitting 1:3–1:20. Suspension cell lines were passaged at least once a week by 1:5–1:50 dilution. Melanoma lines from patients MM909.06 (R10), MM909.11 (R10), MM909.14 (R10), MM909.15 (R10), MM909.22 (R10), MM909.24 (R10), and MM909.46 (D10-F12) were generated from metastatic lesions at the CCIT-DK. Sarcoma line SAR26 from patient SAR26 was also generated at the CCIT-DK and cultured in D10-F12. FM-6 (R10), FM-72 (D10-F12) and FM-82 (R10) were provided by the CCIT-DK. Melanoma SK-MEL-28 was sourced locally and cultured in R10. Acute myeloid leukaemia lines OCI-M2 (R10), MONO-MAC-1 (R10 with 1 mM sodium pyruvate and 1X MEM non-essential amino acids) and Kasumi-6 (R10) were purchased from DSMZ (Braunschweig, Germany). Pancreatic carcinoma AsPC-1 was obtained from ATCC and cultured in R10 as adherent cells. Cervical cancer line SiHa was sourced locally and cultured in D10-F12. E6.1 Jurkat cells were purchased from the ATCC and cultured in R10 as suspension cells and passaged 1:2–1:20 one to three times a week.

**Table 2:** Cancer cell lines used in this study and their HLA class I genotypes.

Cell line	HLA-A*	HLA-B*	HLA-C*	Cancer type
FM-6	01:01 02:01	07:02 08:01	07:01 07:02	Melanoma
FM-74	31:01 24:02	15:01 35:01	03:03 04:01	Melanoma
FM-82	01:01 02:01	08:01 15:01	03:04 07:04	Melanoma
MEL624	02:01 03:01	07:02 14:02	07:02 08:02	Melanoma
MM909.06	02:01 30:01	13:02 15:01	03:04 06:02	Melanoma
MM909.11	01:01 03:01	15:01 40:01	03:04	Melanoma
MM909.14	26:01 08:01	40:01	03:04 07:02	Melanoma
MM909.15	03:01 31:01	08:01 44:02	07:01	Melanoma
MM909.22	01:01 08:01	44:02	05:01 07:01	Melanoma
MM909.24	02:01 30:02	40:01	03:04	Melanoma
MM909.46	03:01 23:01	18:01 44:03	04:01 07:01	Melanoma
SK-MEL-28	11:01	40:01	03:04	Melanoma
SAR26	01:01 02:01	43:01 45:01	02:02 06:02	Myofibroblastic sarcoma
OCI-M2	02:01	08:01 44:02	05:01 07:06	Acute myeloid leukaemia
MONO-MAC-1	03:01	07:02 51:01	07:02 15:02	Acute myeloid leukaemia
Kasumi-6	02:01 31:01	15:01 51:01	03:04 14:02	Acute myeloid leukaemia
AsPC-1	01:01 26:01	15:01	03:03 03:04	Pancreatic
SiHa	24:02	40:02	03:04	Cervical

### Flow cytometry

Antibodies, viability stain and human FcR blocking reagent information are detailed in Table 3. Data were acquired on a BD FACS Canto (BD Biosciences, Franklin Lakes, NJ, USA) or ACEA NovoCyte 3005 flow cytometer equipped with a NovoSampler Pro (ACEA, Agilent, Santa Clara, CA, USA). Cell sorting was performed on a BD FACS Aria III (BD Biosciences) or SONY MA900 multi-application cell sorter (SONY Biotechnology, San Jose, CA, USA).

**Table 3:** Viability stain, FcR block, and antibody details.

Antibody or reagent	Conjugate/channel	Clone	Supplier	Stain/label volume	Per stain/label
ViViD viability stain	Pacific Blue channel	—	Thermo Fisher Scientific	50 μl	1:2000
Human FcR block	—	—	Miltenyi Biotec	50 μl	5 μl
TNF	PE-Vio 770	cA2	Miltenyi Biotec	100 μl	1:100
CD107a	PE	H4A3	BD Biosciences	100 μl	1:100
CD3	PerCP	UCHT1	BioLegend	50 μl	2 μg/ml
αβ TCR	FITC or PE	IP26	BioLegend	50 μl	4 μg/ml
γδ TCR	APC	REA591	Miltenyi Biotec	50 μl	1:50
CD8	APC-Vio 770	BW135/80	Miltenyi Biotec	50 μl	1:100
CD4	BV510	OKT4	BioLegend	50 μl	2 μg/ml
HLA-A*02	APC	BB7.2	BioLegend	50 μl	2 μg/ml
β2M	APC	2M2	BioLegend	50 μl	0.1 μg/ml
HLA class I	APC	W6/32	BioLegend	50 μl	1.2 μg/ml
HLA class II	FITC	Tü39	BioLegend	50 μl	4 μg/ml
rCD2	PE or FITC	OX-34	BioLegend	50 μl	2 μg/ml (PE) 5 μg/ml (FITC)
CD69	APC	FN50	BioLegend	50 μl	2 μg/ml
Vδ1	FITC	REAL277	Miltenyi Biotec	50 μl	1:50

Antibody concentrations are reported either as dilution factors or as µg/ml, depending on the information provided by the manufacturer: Thermo Fisher Scientific (Waltham, Massachusetts, USA), Miltenyi Biotec (Bergisch Gladbach, Germany), and BioLegend (San Diego, CA, USA).

### T107 assay

T-cells were rested in R5 medium (as for R10, with 5% FBS) for 24 h prior to assay setup to reduce spontaneous activation. T-cells were rested in plate formats matched to cell number: 0.1–0.2 × 10^6^ cells/well (96-well U-bottom, 200 μl), 1–2 × 10^6^ cells/well (48-well, 1 ml), or 3–4 × 10^6^ cells/well (24-well, 2 ml). For the assay, T-cells (3 × 10^4^) were co-incubated with target cells (6 × 10^4^) per well of a 96 U well plate, for 4 h at 37°C in the presence of 30 μM TNF-processing inhibitor TAPI-0 (Santa Cruz Biotechnology, Dallas, TX, USA) in 100 μl of R5, together with antibodies TNF PE-Vio 770 and CD107a PE. Incubation time of 3–6 h were performed during the optimization phase of the study, with 4 h adopted as the standard incubation time. Following co-incubation, cells were washed three times with PBS and stained with LIVE/DEAD fixable Violet dead cell stain Viability Dye (ViViD) for 5 min at room temperature, followed by human FcR receptor blocking reagent for 10 min at room temperature. Without washing surface staining antibodies were added: CD3 PerCP, CD8 APC-Vio 770, CD4 BV510, αβ TCR FITC, and γδ TCR APC, and incubated for 20 min on ice. Cells were washed three times with PBS and placed on ice until flow cytometry. In some cases the cells were fixed, using 2% formaldehyde for 20 min on ice, followed by three washes with PBS, then acquisition within 24 h.

### Intracellular cytokine staining assay

T-cells were rested, and cell numbers and densities were the same as those used for the T107 assay. At the start of the assay, CD107a antibody was added in the presence of GolgiStop and GolgiPlug (both BD Biosciences), according to the manufacturer’s instructions. Following incubation, cells were stained with ViViD and antibodies as for the T107 assay. Cells were then incubated with Cytofix/Cytoperm^TM^ (BD Biosciences) for 20 min according to the manufacturer’s instructions, including washing with Perm/Wash^TM^ (BD Biosciences), followed by intracellular staining for 20 min on ice with TNF PE-Vio 770 antibody. Cells were washed once with Perm/Wash^TM^, then twice with PBS, before flow cytometry within 24 h.

### αβ T-cell receptor sequencing

Flow-sorted T-cell populations, or T-cell clones from culture, were stabilized in RNAprotect Cell Reagent (Qiagen, Venlo, The Netherlands). TCR sequencing was performed as described previously [41]. Briefly, RNA was extracted using the RNeasy Micro Kit (Qiagen), and cDNA was generated with the 5′/3′ SMARTer kit (Takara Bio, Kusatsu, Shiga, Japan) using MMLV reverse transcriptase, a 3′ oligo-dT primer, and a 5′ universal anchor oligonucleotide. Step-Out PCR was carried out with *TRAC*/*TRBC*-specific reverse primers (Eurofins Genomics, Ebersberg, Germany) and a universal anchor-specific forward primer (Takara Bio) using Phusion High-Fidelity polymerase (Thermo Fisher Scientific, Waltham, MA, USA) under the following conditions: 94°C for 5 min; 30 cycles of 94°C for 30 s, 63°C (α) or 66°C (β) for 30 s, and 72°C for 120 s. Step-Out PCR products (5 μl) were used for nested PCRs, with barcoded universal forward and TRAC/TRBC reverse primers under similar cycling conditions (final extension 72°C, 10 min). Amplicons were resolved on 1% agarose gels, purified (Monarch®, New England Biolabs, Ipswich, MA, USA), and sequenced on an Illumina MiSeq using v2 chemistry. Sequences were processed using MiXCR v3.0.7. Clonotypes representing <5% of reads were excluded, and percentages were recalculated after filtering.

### Gene knockout in cancer cells

Guide RNAs targeting HLA-A*02:01 (5′-CCAGAGCCCCCGAGAGTAGC-3′) and β2-microglobulin (5′-ACTCACGCTGGATAGCCTCC-3’) were designed and cloned into the pLentiCRISPR v2 vector with a puromycin resistance gene (Addgene plasmid # 52961; kindly provided by Dr Feng Zhang). Melan-A knockout MM909.24 melanoma cells were generated as previously described [42]. Lentiviral particles were generated using 3.3 μg of gRNA-containing pLentiCRISPR v2 transfer vector, 0.825 μg of pMD2.G envelope plasmid (gift from Didier Trono, Addgene plasmid # 12259) and 1.65 μg of psPAX2 packaging plasmid (Addgene plasmid # 12260), mixed in 300 μl of Opti-MEM, reduced serum medium (Thermo Fisher Scientific) followed by mixing with 1 μg/ml Polyethylenimine (PEI; Merck Group) at a 3:1 PEI: plasmid ratio. Plasmid/PEI mixtures were incubated at room temperature for 15 min, added dropwise to HEK293T cells (80% confluence in one well of a six-well plate) and incubated at 37°C in a 5% CO_2_ humidified atmosphere. Culture media was changed after 24 h. Supernatants were harvested at 36 and 48 h post-transfection, centrifuged (400×g for 5 mins) and 0.45 μm filtered and used immediately or stored at −80°C. Viral transduction was performed by spin-infection at 400×g for 2 h with 8 μg/ml polybrene (Sigma Aldrich) and incubated at 37°C and 5% CO_2_, followed by a media change after overnight incubation. Knockout was assessed via antibody staining, and knockout cells enriched by flow cytometry sorting and cloned by limiting dilution in 96 well plates (U-well for suspension cells, and flat-well for adherent cells) with 0.5, 1 or 3 cancer cells per well, and 5 × 10^4^ per well of irradiated PBMCs from three donors. Clones arising from wells seeded at the lowest cell density were selected for validation by antibody staining.

### Killing assays

Chromium cytotoxicity assays were performed as described previously [41]. For flow cytometry-based assays, target cells were co-incubated with and without T-cells at E:T ratios and duration indicated in the results, in 96U-well plates containing media of 50:50 T-cell media with 20 IU/ml IL-2 and IL-15 and the targets cells preferred media. On the day of harvest, 2 × 10^4^ C1R cells were labelled with 0.1 μM CFSE (Thermo Fisher Scientific) and added to each assay well, followed by incubation with FcR blocking reagent and staining with ViViD, CD3 PerCP and CD8 APC-Vio 770 antibodies. T-cells and dead/dying cells were excluded from analysis, leaving viable target cells and CFSE^+^ C1Rs to calculate the percentage of killing using the following equation.


$$\mathrm{killing}=100\;\text{-}\left(\left(\frac{\mathrm{with}\;T\;\mathrm{cells}:\;\text{\%}\;\mathrm{target}\;\mathrm{cells}÷\text{\%}\;C1R\;\mathrm{CFSE}}{\mathrm{without}\;T\;\mathrm{cells}:\;\text{\%}\;\mathrm{target}\;\mathrm{cells}÷\text{\%}\;C1R\;\mathrm{CFSE}}\right)\times 100\right)$$

### Peptide assays

Single-peptide and positional scanning combinatorial peptide library (PS-CPL) screens and assays were performed as previously described, using M1P-1β as the functional readout, measured by ELISA [43]. The tumour-associated antigen database CANTiGEN was used to identify the TAG peptide for clone GD-22.

### γδ T-cell receptor pairing by iPair^TM^

Cancer-reactive γδ TCR^+^ cells identified by the T107 assay were single-cell sorted directly into iCapture plates using a BD Aria cell sorter (one cell per well). Plates were frozen at −80°C and shipped to iRepertoire (Huntsville, AL, USA) for paired TCR sequencing.

### TMA γδ TCR transduced Jurkat cells and CD69 assays

The TMA TCR was expressed in E6.1 Jurkat TCR knockout cells. Transfection and lentiviral transduction as previously described [41]. For TCR knockout, oligonucleotides for the sgRNA sequence of TRBC1 and TRBC2 genes (5ʹ-CAAACACAGCGACCTCGGGT-3ʹ) [44] were designed and cloned into the pLentiCRISPR v2 plasmid with a puromycin resistance gene (Addgene plasmid # 52961; kindly provided by Dr Feng Zhan). To assess TCR knockout, cells were washed three times with PBS followed by staining with ViViD for 5 min at room temperature, and without washing stained with αβ TCR PE antibody, then fixed as above. Codon-optimized TMA TCR was expressed from the pSF plasmid, with the TRG and TRD chains separated by a ‘self-cleaving’ T2A, and P2A sequence between the TRD gene and rCD2 co-marker: XbaI-Kozak-TRG-T2A-TRD-XhoI-P2A-rCD2-SalI-Stop. Cells were stained as described above, using rCD2 PE and Vδ1 FITC antibodies to assess TCR expression. Jurkat cells were enriched for TCR expression using the rCD2 PE co-marker antibody and anti-PE magnetic microbeads according to the manufacturer’s instructions (Miltenyi Biotec, Bergisch Gladbach, Germany).

CD69 assays were performed as previously described [41]. Phorbol 12-Myristate 13-Acetate (Promega, Wisconsin, USA) (0.02 μg/ml) was used as positive control for CD69 expression. Cells were stained with rCD2 PE (TCR co-marker) and CD69 APC antibodies. Gating for Jurkat cells: (FSC-H versus SSC-H), single cells (FSC-A versus FSC-H), viable cells (SSC-H vs ViViD stain low/negative), rCD2 (for TCR expression), then CD69 APC, from which mean fluorescence intensity values were obtained.

### TMA γδ TCR-T

The TMA TCR pSF plasmid was used, as described above. Transfection and transduction as previously described [41]. TCR replacement was achieved using the sgRNA as above and previously described [41, 44]. TCR staining as above for Jurkat cells, using ViViD, and CD3 PerCP, CD8 APC-Vio 770, rCD2 PE, Vδ1 FITC antibodies. T107 assays were set up as above using TNF PE-Vio 770 and CD107a FITC antibodies added at the start of the assay, followed by staining with ViViD, CD3 PerCP, CD8 APC-Vio 770 and rCD2 PE.

### Software and statistics

Unless stated otherwise, all data were displayed using GraphPad Prism software and analysed using Microsoft Excel. Flow cytometry data analysed with FlowJo software (Tree Star Inc., Ashland, Oregon, US). The graphical abstract and Supplementary Figure S1 was created using BioRender (BioRender.com)

## Results

### The T107 assay enables rapid identification and isolation of cancer-reactive T-cells without prior antigen knowledge

TILs represent a key source of cancer-reactive T-cell receptors. TCRs derived from patients who have achieved complete and durable remission of metastatic disease following TIL therapy are of particular interest, as they can exhibit unconventional specificities and mechanisms of tumour recognition [42]. Metastatic melanoma patient MM909.24 underwent complete durable remission following treatment with TILs at the CCIT-DK [5] and remains disease free over 12 years after treatment. Patient characteristics and cancer cell lines used in this study are summarized in Tables 1 and 2.

**Figure 1:**
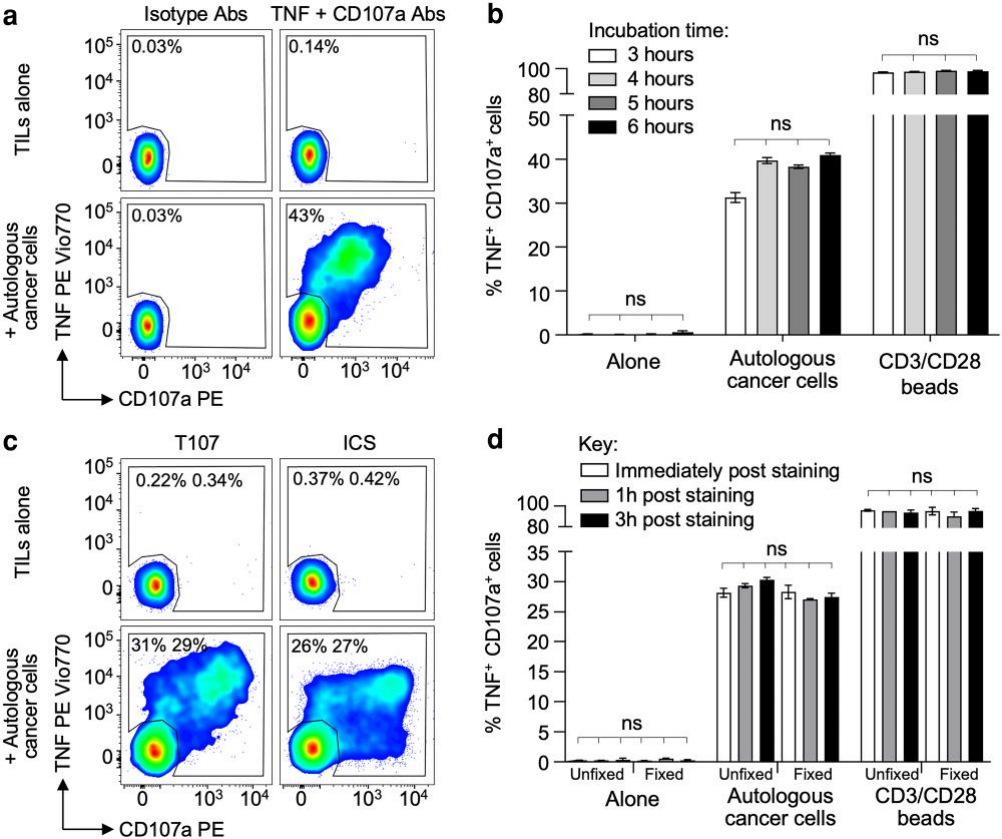
Reliable detection of TNF and CD107a on activated cells using the T107 assay. (a) Tumour-infiltrating lymphocytes (TILs) from patient MM909.24 were co-incubated with autologous melanoma cells for 4 h using the T107 assay. Isotype control antibodies confirm specific surface detection of CD107a and tumour necrosis factor (TNF) on activated T-cells. (b) MM909.24 TILs were incubated with or without autologous melanoma cells, with CD3/CD28 Dynabeads used as a positive control. T107 assays were performed for the incubation times indicated. No significant difference in the frequency of reactive T-cells was observed across the time points tested, and 4 h was adopted as the standard T107 assay duration. (c) Comparison of the T107 assay and intracellular cytokine staining (ICS) using MM909.24 TILs incubated with or without autologous melanoma cells. The T107 assay revealed a brighter TNF-positive population compared with ICS. Percentages of TNF^+^ CD107a ^+^ cells are shown within the indicated gates. Performed in duplicate. (d) Stability of TNF and CD107a staining on unfixed reactive T-cells following the T107 assay. MM909.24 TILs were incubated with autologous melanoma cells or CD3/CD28 Dynabeads for 4 h, stained with antibodies, and either left unfixed or fixed before storage on ice for 1 or 3 h as indicated. TNF and CD107a staining remained stable under these conditions, supporting downstream sorting of viable cells. Performed in duplicate.

To enable rapid identification and recovery of viable cancer-reactive T-cells without *a priori* knowledge of antigen specificity, we employed a flow cytometry-based assay that detects surface mobilization of CD107a [45] and capture of tumour necrosis factor (TNF) [46] following short-term co-culture with cancer cells, referred to here as the T107 assay (schematized in the Graphical Abstract and Supplementary Figure S1; gating strategy shown in Supplementary Figure S2a). Following 4 h co-incubation of MM909.24 TILs with the autologous melanoma cell line, a discrete population of CD107a^+^ TNF^+^ T-cells was detected, with specificity confirmed using matched isotype control antibodies (Fig. 1a). The frequency of responding cells increased rapidly and plateaued within 4 h of incubation, with no further increase observed at later time points (Fig. 1b and Supplementary Figure S2b). Based on these findings, a 4 h incubation was used as the standard assay duration throughout this study unless otherwise stated.

We next compared the performance of the T107 assay with optimized intracellular cytokine staining (ICS) for TNF and CD107a using monensin, brefeldin A, and commercially available fixation and permeabilization reagents containing formaldehyde and saponin. Under matched conditions, the T107 assay yielded a brighter TNF^+^ population than ICS (Fig. 1c and Supplementary Figure S2c), facilitating clearer discrimination of responding cells. From a practical perspective, TNF and CD107a surface staining remained stable on unfixed cells for up to 3 h when samples were maintained on ice, with no appreciable loss of signal compared to fixed samples (Fig. 1d and Supplementary Figure S3a). This stability, combined with the absence of fixation or intracellular staining requirements, renders the T107 assay compatible with downstream sorting and recovery of viable cancer-reactive T-cells for functional and molecular analyses.

### The T107 assay detects cancer-reactive αβ and γδ T-cell receptor subsets across CD4 and CD8 phenotypes

**Figure 2:**
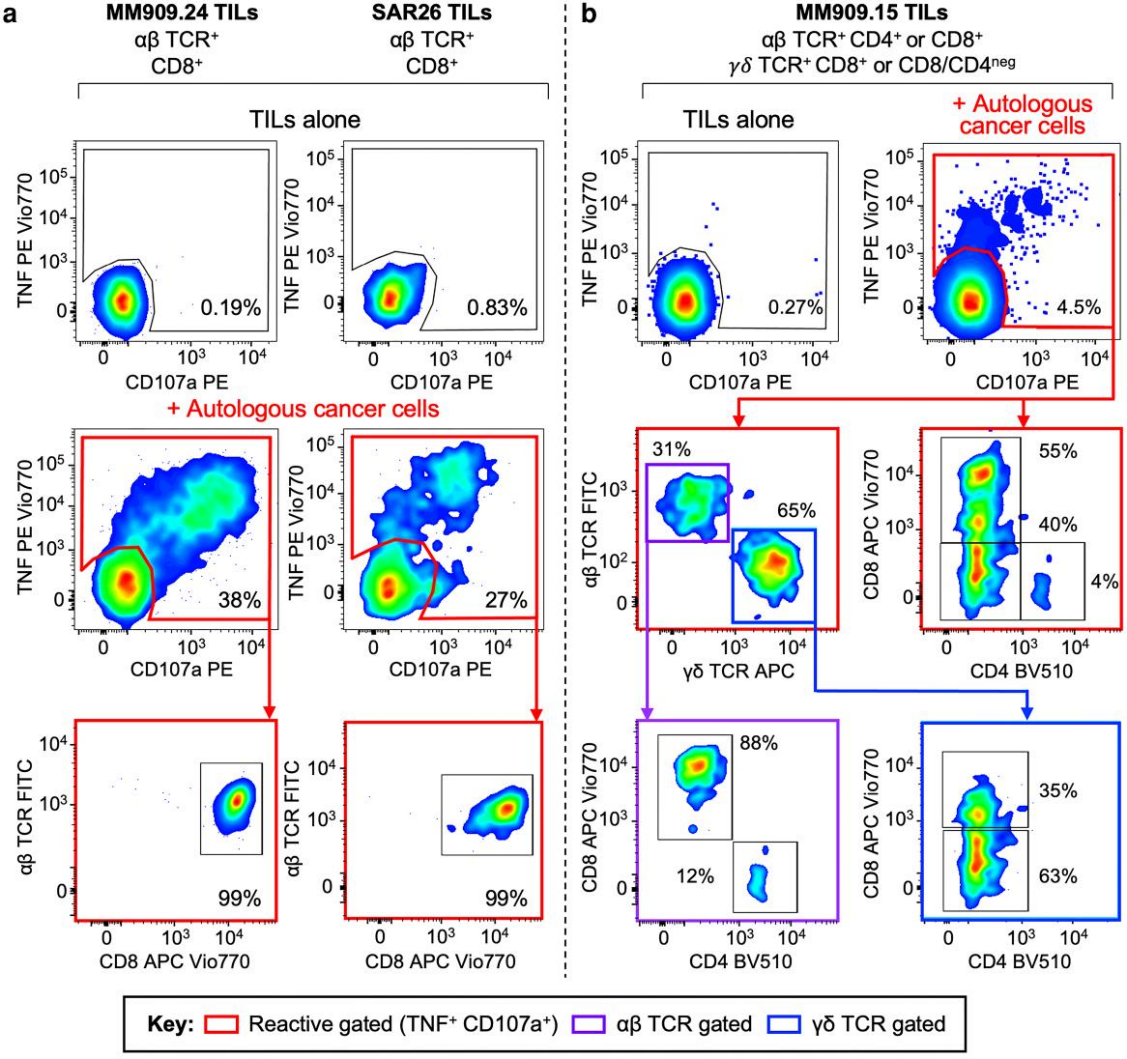
The T107 assay readily detects activated αβ TCR+, γδ TCR+, CD8^+^, and CD4^+^ T-cell subsets. Tumour-infiltrating lymphocytes (TILs) from three cancer patients were incubated with their respective autologous cancer cells for 4 h using the T107 assay, followed by surface staining to assess T-cell receptor expression (αβ and γδ) and co-receptor (CD4 and CD8) expression. (a) TILs from melanoma patient MM909.24 and sarcoma patient 26 (SAR26) were dominated by αβ TCR^+^ CD8^+^ cancer-reactive T-cells. Cancer reactive T-cells were defined as TNF^+^/CD107a^+^. (b) TILs from melanoma patient MM909.15 contained multiple cancer-reactive T-cell subsets, including αβ TCR+ and γδ TCR+ T-cell populations within both CD8^+^ and CD4^+^ compartments. Cancer reactive T-cells were defined as TNF^+^/CD107a^+^. Functional responses were analysed by both TCR lineage (αβ TCR+ or γδ TCR+, with subsequent CD8^+^/CD4^+^ subdivision) and by direct CD8^+^/CD4^+^ analysis from the TNF^+^/CD107a^+^ population. Percentages are displayed within or adjacent to the gated populations.

T107 analysis of TILs from melanoma patient MM909.24 and sarcoma patient SAR26 revealed that approximately 38% and 27% of the respective TIL products responded to co-culture with the autologous tumour line, with the majority of cancer-reactive T-cells having a CD8^+^ αβ TCR+ phenotype (Fig. 2a). The absence of detectable CD4^+^ cancer-reactive T-cell populations in these samples may reflect the lack of major histocompatibility complex (MHC) class II expression by the corresponding autologous tumour cell lines (Supplementary Figure S3b). In contrast, TILs derived from melanoma patient MM909.15, who underwent complete remission following TIL therapy, contained a broader spectrum of cancer-reactive T-cell subsets (Fig. 2b). Approximately 4.5% of the TIL population responded to co-culture with the autologous melanoma cell line. Of these responding cells, around 31% expressed an αβ TCR, with a minority of this subset expressing the CD4 co-receptor rather than CD8 (Fig. 2b, purple gating). The autologous melanoma cell line from patient MM909.15 expressed MHC class II (Supplementary Figure S3b), raising the possibility that these CD4-positive T-cells directly recognize MHC class II-restricted tumour antigens. Notably, the majority of cancer-reactive T-cells identified in the MM909.15 TIL population expressed a γδ TCR (Fig. 2b, turquoise gating). This γδ T-cell population was predominantly CD4- and CD8 double-negative, although approximately one-third of the responding γδ T-cells expressed the CD8 co-receptor. Furthermore, the TIL from patient MM909.15 contains multiple populations with differing TNF/CD107a intensities (Fig. 2b), suggesting heterogeneity in the responding population into TNF/CD107a low, medium, and high subsets (Supplementary Figure S4a). We observed that αβ TCR^+^ and CD8^+^ T-cells increased in frequency across these subsets, whereas γδ TCR^+^ and CD4^+^ T-cells were most abundant within the TNF/CD107a low population (Supplementary Figure S4b). Notably, CD4^+^ T-cells were largely absent from the TNF/CD107a high subset. However, these data represent a single 4 h snapshot acquired under assay conditions optimized primarily for detection of CD8^+^ αβ TCR+ T-cell responses. Consequently, the relative distribution of γδ TCR+ and CD4^+^ T-cells across the TNF/CD107a gates may reflect differences in activation kinetics and/or functional programme rather than reduced tumour reactivity. Together, these data demonstrate that the T107 assay enables detection and isolation of heterogeneous cancer-reactive αβ and γδ TCR-expressing T-cells across a range of CD4 and CD8 co-receptor phenotypes, without prior knowledge of antigen specificity.

### The T107 assay enriches for cancer-reactive T-cell receptors from diverse tumour-infiltrating lymphocyte repertoires

**Figure 3:**
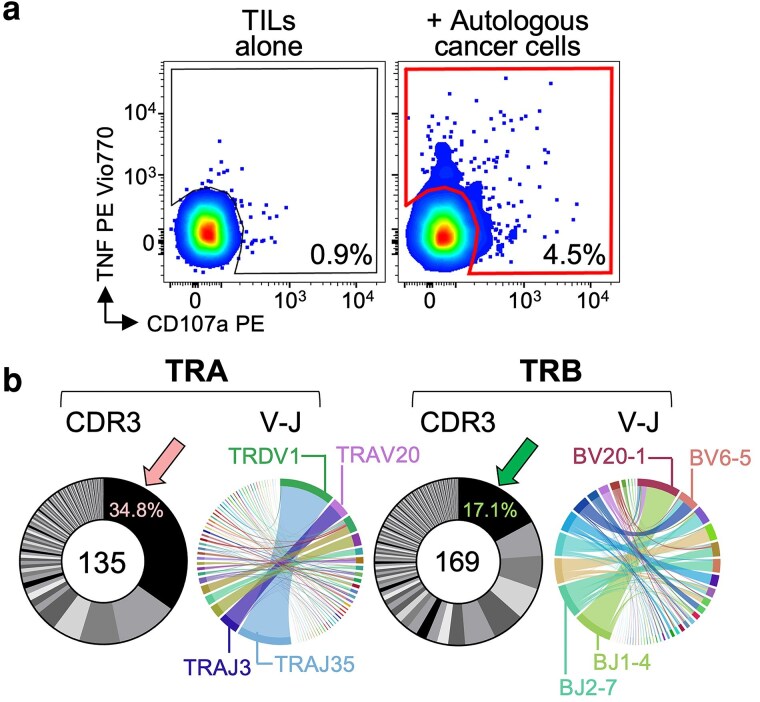
The T107 assay reveals a dominant cancer-reactive clonotype from polyclonal tumour-infiltrating lymphocytes. (a) Tumour-infiltrating lymphocytes (TILs) from metastatic melanoma patient MM909.46 were incubated with or without autologous melanoma cells for 4 h using the T107 assay. Cancer-reactive T-cells were identified as TNF^+^/CD107a^+^ cells and isolated by cell sorting. (b) T-cell receptor sequencing of sorted cancer-reactive T-cells, showing TCR alpha (TRA) and beta (TRB) repertoires. Complementarity-determining region 3 (CDR3) sequences are displayed as pie charts, with each segment representing a unique CDR3 and the total number of detected CDR3s indicated at the centre of each chart. Variable (V) and joining (J) gene usage is shown as Circos plots.

TILs from metastatic melanoma patient MM909.46 exhibited 4.5% reactivity towards autologous melanoma cells in the T107 assay (Fig. 3a). To assess the diversity and relative abundance of cancer-reactive TCR chains, we performed TCR α and β sequencing on the T107-sorted cancer-reactive population as well as on the corresponding bulk, unsorted TILs. Analysis of TCRα chains identified 135 unique CDR3 sequences within the T107-sorted population, compared with 978 unique CDR3α sequences in the bulk TIL repertoire. Within the T107-sorted cancer-reactive population (red gate Fig. 3a), a single dominant CDR3α accounted for 34.8% of all α chain sequences (Fig. 3b). In contrast, this same CDR3α sequence represented only 0.13% of the total CDR3α sequence in the bulk TIL repertoire, indicating a substantial enrichment by the T107 assay. A similar pattern was observed for TCRβ chains. The dominant CDR3β sequence comprised 17.1% of the T107-sorted cancer-reactive population, compared with just 0.35% of total CDR3β sequences in the bulk TILs. Together, these data demonstrate that the T107 assay enriches for dominant cancer-reactive TCR α and β chains from highly diverse TIL repertoires, enabling identification of functionally relevant TCRs that are present at very low frequencies in bulk TILs.

### T107 assay is suitable for parallel sorting of viable T-cells for sequencing and generation of T-cell clones

**Figure 4:**
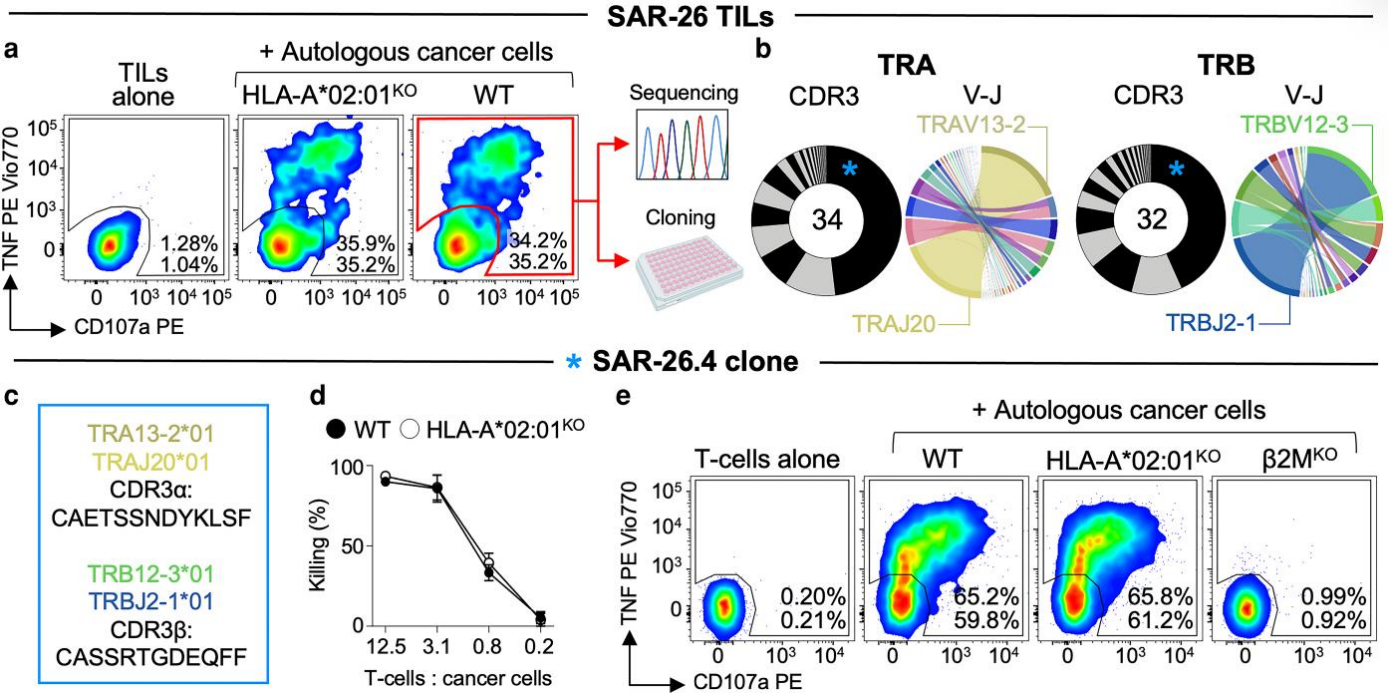
Parallel phenotyping, T-cell receptor sequencing and functional characterization of cancer-reactive T-cells following the T107 assay. (a) Tumour-infiltrating lymphocytes (TILs) from sarcoma patient SAR26 were incubated with the autologous sarcoma cell line for 4 h using the T107 assay, followed by surface phenotyping and sorting of CD107a^+^ TNF^+^ T-cells for downstream TCR sequencing and T-cell cloning. (b) TCR sequencing of T107-sorted cancer-reactive T-cells. TCR alpha (TRA) and beta (TRB) CDR3 sequences are displayed as pie charts, with each segment representing a unique CDR3 and the total number of detected CDR3s indicated at the centre of each chart. Variable (V) and joining (J) gene usage is shown as Circos plots. Asterisks indicate the TRA and TRB chains corresponding to the SAR26.4 CD8-positive T-cell clone. (c) TRA and TRB gene usage and CDR3 sequences of the SAR26.4 T-cell clone derived from the T107-sorted population. (d) Cytotoxic activity of the SAR26.4 T-cell clone against autologous sarcoma cells assessed by chromium-51 release assay. Comparable killing of wild-type and HLA-A*02:01-knockout autologous sarcoma cells indicates that tumour recognition by SAR26.4 is independent of HLA-A*02:01. (e) Recognition of autologous sarcoma cells by SAR26.4 is dependent on β2-microglobulin (β2M). T107 assays were performed using wild-type, HLA-A*02:01-knockout and β2M-knockout autologous sarcoma cells, demonstrating loss of reactivity in the absence of β2M.

The TIL from sarcoma patient SAR26 were co-cultured with the autologous sarcoma cell line for 4 h using the T107 assay. A population of responding T-cells upregulated both CD107a and TNF (Fig. 4a) and was predominantly αβ TCR^+^ and CD8^+^ (Fig. 2a). The SAR26 TILs responded at comparable levels to wild-type autologous sarcoma cells and to an HLA-A*02:01 knockout variant generated by CRISPR-Cas9 (Supplementary Figure S5a; Fig. 4a), suggesting that tumour recognition by the bulk TIL population was not restricted by this patient HLA allele (HLA typing shown in Table 2). TCR sequencing of the T107-sorted responding population revealed more than 30 distinct αβTCRs, with a single α and β chain pair accounting for nearly half of the cancer-reactive population (Fig. 4b). Single-cell sorting and expansion of T107-positive T-cells yielded the SAR26.4 T-cell clone, which was confirmed to be αβ TCR^+^ and CD8^+^ (Supplementary Figure S5b). The SAR26.4 clone expressed a TCR comprising TRAV13-2*01 paired with TRAJ20*01 (CDR3α: CAETSSNDYKLSF) and TRBV12-3*01 paired with TRBJ2-1*01 (CDR3β: CASSRTGDEQFF), corresponding to the dominant TCR chains identified in the T107-sorted population (Fig. 4c). The SAR26.4 T-cell clone efficiently killed autologous sarcoma cells lacking HLA-A*02:01 (Fig. 4d), indicating that tumour recognition by this clone is independent of this HLA allele. In contrast, deletion of β2-microglobulin from autologous sarcoma cells by CRISPR-Cas9 (Supplementary Figure S5a) abrogated SAR26.4 reactivity (Fig. 4e), demonstrating dependence on HLA class I expression or other β2M-containing presentation platform. Together, these data show that the T107 assay supports parallel phenotyping, receptor sequencing, and isolation of viable cancer-reactive T-cells, enabling direct generation of T-cell clones for functional characterization and determination of HLA restriction.

### The T107 assay enables isolation of orphan T-cell receptor clonotypes for epitope discovery

**Figure 5:**
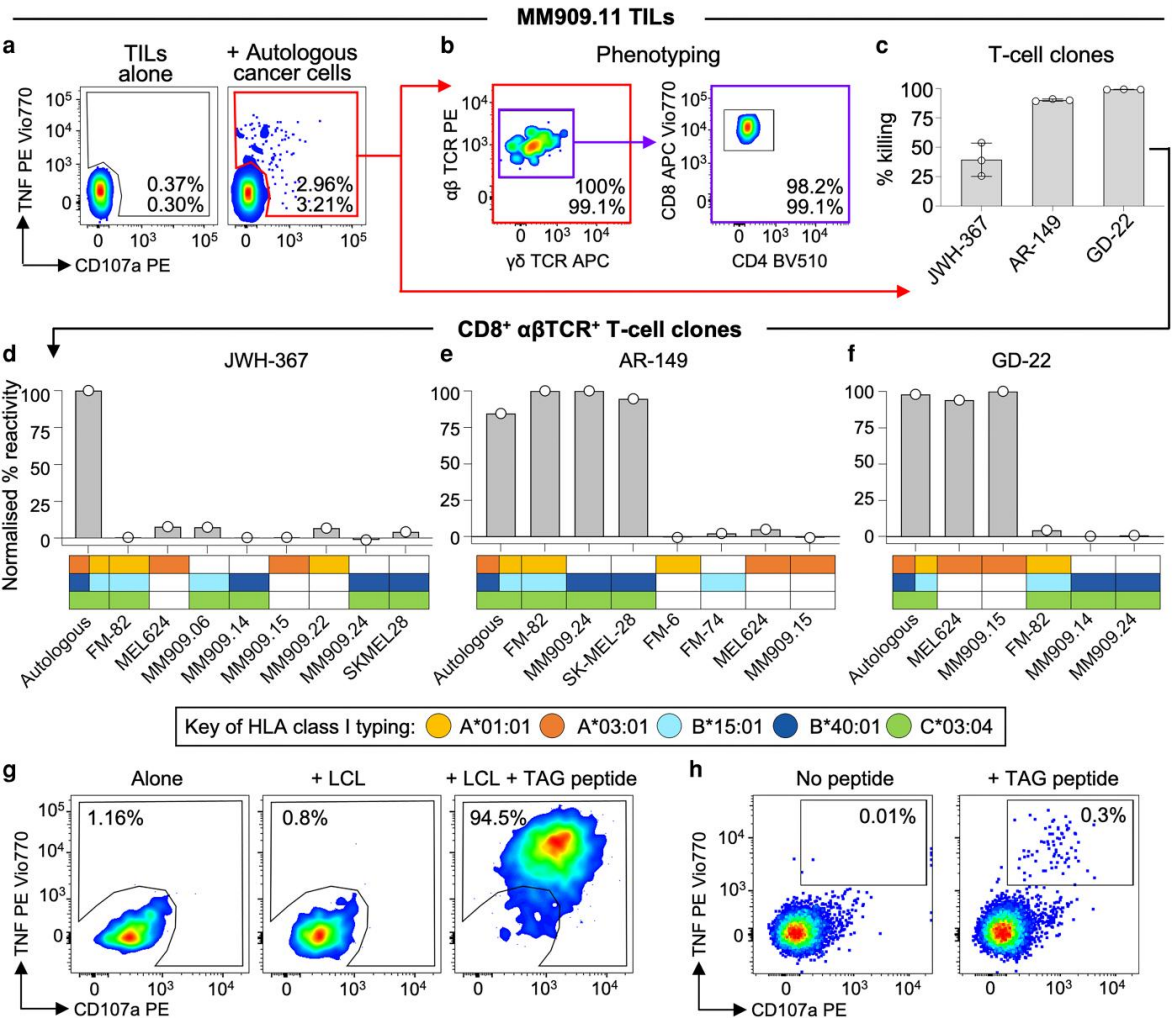
Characterization of tumour-infiltrating lymphocyte derived cancer-reactive T-cell clones procured using the T107 assay. (a) TILs from melanoma patient MM909.11 were incubated with the autologous melanoma cell line for 4 h using the T107 assay. Percentages of CD107a^+^ TNF^+^ cells are shown for duplicate assay conditions. (b) Phenotypic characterization of the cancer-reactive T-cell population identified in A. Percentages are shown for duplicate conditions. (c) T-cell clones were generated from the cancer-reactive population identified in A. CD8^+^ αβ TCR^+^ T-cell clones were assessed in an overnight cytotoxicity assay against autologous melanoma cells. (d–f) Cancer reactivity of CD8^+^ T-cell clones JWH-367 (d), AR-149 (e), and GD-22 (f) assessed by the T107 assay following 4 h co-culture with autologous melanoma cells and a panel of partially HLA-matched melanoma cell lines. The HLA class I types of the autologous and non-autologous melanoma cell lines are indicated on the *x*-axis and in the accompanying key. (g) Reactivity of the GD-22 T-cell clone to the TAG-derived peptide RLSNRLLLR presented by autologous lymphoblastoid cell line (LCL). (h) Reactivity of bulk TILs from patient MM909.11 to the TAG-derived peptide RLSNRLLLR.

Viable cancer-reactive T-cell clones were generated using the T107 assay from TILs derived from metastatic melanoma patient MM909.11, who achieved a complete and durable response following TIL therapy (Table 1). In a 4 h T107 assay, ∼3.1% of this TIL product responded to co-culture with the autologous melanoma cell line (Fig. 5a), and more than 99% of responding cells were αβ TCR^+^ and CD8^+^ (Fig. 5b). Cancer-reactive T-cells were sorted and cloned, yielding three αβ TCR^+^ CD8^+^ T-cell clones (JWH-367, AR-149 and GD-22) for further analysis. All three clones killed autologous melanoma cells (Fig. 5c). To assess the breadth and restriction of tumour recognition, the clones were tested by T107 assay against a panel of melanoma cell lines, including the autologous tumour and additional melanomas partially matched for HLA class I alleles expressed by patient MM909.11 (Fig. 5d–f and Table 2).

The JWH-367 T-cell clone responded exclusively to the autologous melanoma cell line and did not recognize other melanoma cell lines expressing any of the patient’s HLA class I alleles (Fig. 5d and Supplementary Figures S6a and b). Deletion of β2M from the autologous melanoma cells abrogated JWH-367 reactivity (Supplementary Figure S6c), indicating likely HLA class I dependence. Together, these findings suggest that JWH-367 recognizes a patient-specific, HLA-restricted neoantigen.

The AR-149 T-cell clone recognized melanoma cell lines expressing HLA-C*03:04, an HLA class I allele homozygously expressed by the autologous tumour (Fig. 5e and Supplementary Figures S7a and b). In contrast, AR-149 did not respond to cervical (SiHa) or pancreatic (AsPC-1) cancer cell lines expressing HLA-C*03:04 (Supplementary Figure S7b), suggesting tumour-type specificity. To assess whether AR-149 recognized a known melanoma-associated antigen, we tested its response to HLA-C*03:04^+^ MM909.24 melanoma cells lacking Melan-A expression, generated by CRISPR-Cas9 knockout [42]. AR-149 recognized wild-type and Melan-A-deficient melanoma cells at comparable levels (Supplementary Figure S7c), indicating that recognition was not mediated by Melan-A-derived peptides. Furthermore, AR-149 did not respond to known HLA-C*03:04-restricted peptide epitopes derived from NY-ESO-1 [47] or MAGE-A1 [48] (Supplementary Figure S7d). Collectively, these data indicate that AR-149 probably recognizes a shared, previously undescribed melanoma-specific epitope presented by HLA-C*03:04.

The third clone, GD-22, preferentially recognized melanoma cell lines expressing HLA-A*03:01 (Fig. 5f and Supplementary Figure S8a). We therefore advanced GD-22 to epitope discovery using the CANTiGEN pipeline, which integrates PS-CPL screening with a curated database of cancer-associated proteins to predict candidate T-cell epitopes [42]. Peptide-length profiling indicated a preference for 9-amino-acid peptides, and a 9-mer PS-CPL screen was performed (Supplementary Figure S8b). The resulting specificity matrix was used to interrogate the CANTiGEN database, identifying a candidate HLA-A*03:01-restricted peptide derived from a cancer/testis tumour antigen gene (TAG), RLSNRLLLR. This peptide activated the GD-22 T-cell clone (Fig. 5g). Finally, stimulation of bulk TILs from patient MM909.11 with the TAG-derived peptide revealed a discrete population of CD107a ^+^ TNF^+^ CD8^+^ T-cells (Fig. 5h), representing approximately 0.3% of the total TIL population and around 10% of the cancer-reactive T-cell compartment identified by the T107 assay (Fig. 5a).

### The T107 assay enables isolation of paired γδ T-cell receptors and generation of cancer-reactive γδ TCR-T cells

TILs from metastatic melanoma patient MM909.15 contained a population of γδ TCR^+^ T-cells that recognized autologous melanoma cells in a β2M-independent manner (Supplementary Figure S9a). To identify γδ TCRs with potential reactivity against additional cancer types, the TIL product from this patient was first enriched by sorting viable CD3 ^+^ TNF ^+^ CD107a^+^ γδ TCR^+^ T-cells following co-culture with autologous melanoma cells. Sorted cells were expanded using allogeneic PBMCs and phytohaemagglutinin.

**Figure 6:**
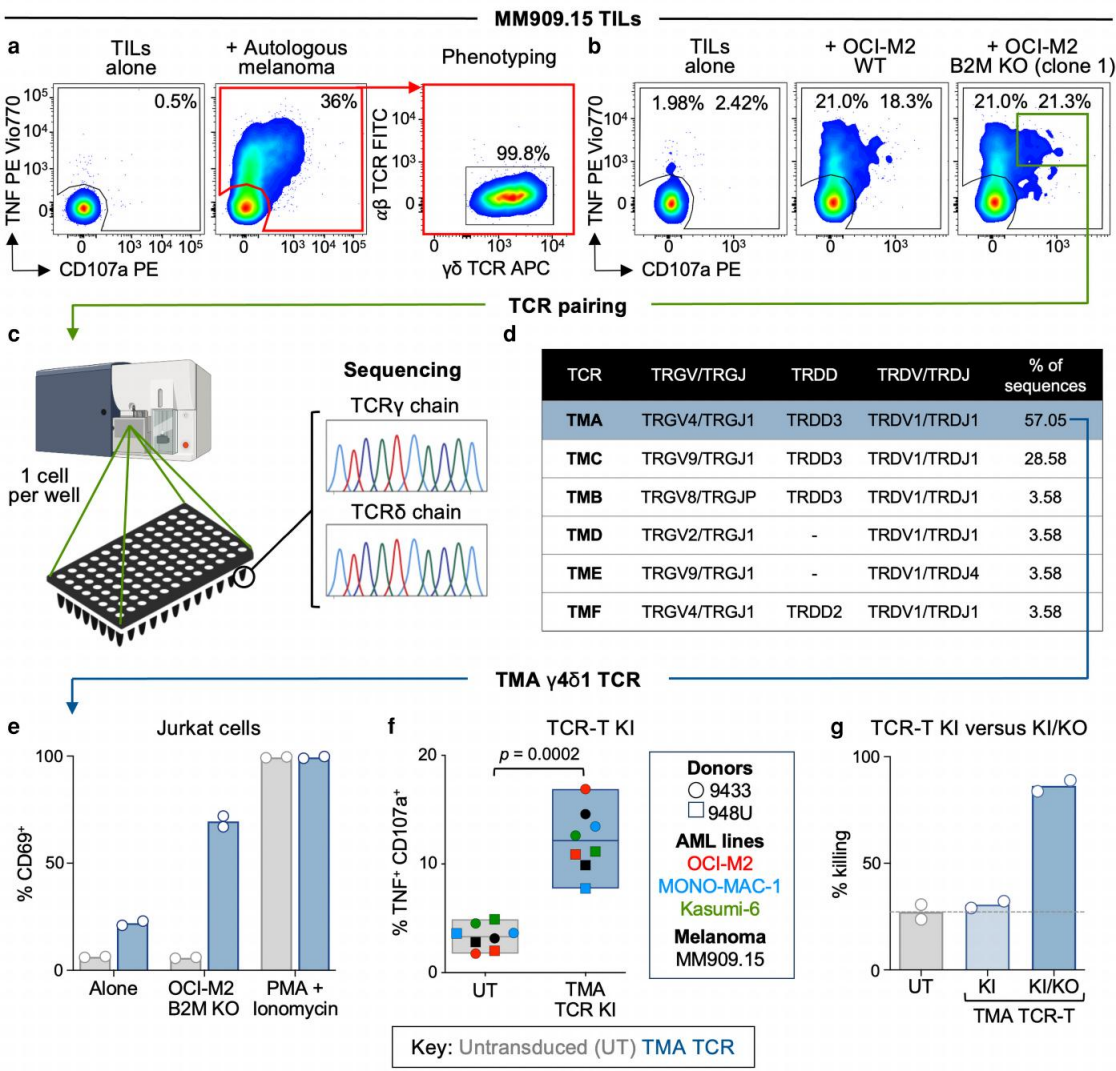
Pairing and functional testing of cancer-reactive γδ TCRs derived from a clinical tumour-infiltrating lymphocyte product. (a) Autologous melanoma reactivity of TILs from metastatic melanoma patient MM909.15 that had been previously enriched for cancer-reactive γδ TCR^+^ cells using the T107 assay and cell sorting. (b) T107 assay performed using the enriched TIL population from A co-cultured with the acute myeloid leukaemia cell line OCI-M2, either wild-type or β2-microglobulin knockout (β2M KO). CD3^+^/TNF^+^/CD107a^+^/γδ TCR^+^ T-cells were identified and sorted from the OCI-M2 β2M KO condition for downstream TCR pairing. (c) Single CD3^+^/TNF^+^/CD107a^+^/γδ TCR^+^ T-cells were sorted into iCapture® plates for paired gamma and delta chain recovery using the iRepertoire® platform. (d) Hierarchy of recovered γδ TCR pairs, showing variable and joining gene usage for γ and δ chains. (e) The dominant γδ TCR pair (TMA) was expressed in Jurkat cells and assessed in an overnight CD69 activation assay using OCI-M2 β2M KO target cells. The TMA TCR reacts to many cancer types and exhibits some baseline reactivity to the host Jurkat cells. PMA and ionomycin were used as positive controls. (f) Primary CD8^+^ T-cells engineered to express the TMA γδ TCR pair by knock-in (KI) were assessed in a 4 h T107 assay against β2M KO AML cell lines and β2M KO autologous melanoma cells. Analysis was gated on viable CD3^+^ CD8^+^ rCD2^+^ T-cells. (g) Primary CD8^+^ T-cells expressing the TMA γδ TCR pair, generated either by knock-in alone (KI) or by TCR replacement (knock-in with endogenous TCR beta chain deletion (KI/KO)), were assessed in an overnight cytotoxicity assay against OCI-M2 target cells.

The enriched γδ TCR^+^ T-cell line exhibited robust reactivity towards autologous melanoma, with approximately 36% of cells expressing TNF and CD107a following a 4 h T107 assay and was more than 99% γδ TCR^+^ (Fig. 6a). This enriched population also responded to the acute myeloid leukaemia cell line OCI-M2, with comparable reactivity towards wild-type and β2 M knockout targets generated by CRISPR-Cas9, indicating β2M-independent recognition (Fig. 6b). Cancer-reactive γδ TCR^+^ T-cells were sorted from the OCI-M2 β2 M knockout condition directly into iCapture plates for paired TCR sequencing using the iRepertoire iPair platform (Fig. 6b and c). Six paired γδ TCRs were identified, utilizing TRGV2, TRGV4, TRGV8, or TRGV9 variable genes paired with TRDV1 (Fig. 6d). More than 57% of recovered pairs comprised a single dominant TRGV4 TRDV1 TCR, designated TMA.

The TMA γδ TCR was expressed in TCR-deficient Jurkat cells, where surface expression was confirmed (Supplementary Figure S9b) and functional activation was observed following co-culture with OCI-M2 β2M knockout cells (Fig. 6e). The TMA TCR was also successfully expressed in primary αβ TCR^+^ CD8^+^ T-cells from two healthy donors (Supplementary Figure S9c) and mediated functional responses in a T107 assay against β2 M knockout MM909.15 melanoma cells and three β2M knockout AML cell lines, OCI-M2, MONO-MAC-1, and Kasumi-6 (Fig. 6f).

Finally, we compared knock-in of the TMA γδ TCR alone with TCR replacement, in which the endogenous TCRβ chain was deleted using CRISPR-Cas9 [44]. TCR replacement resulted in higher surface expression of the TMA TCR (Supplementary Figure S9c) and substantially enhanced cytotoxic activity in an overnight killing assay against OCI-M2 wild-type cells, compared with TMA knock-in alone or untransduced control T-cells (Fig. 6g). Together, these data demonstrate that the T107 assay enables isolation of cancer-reactive γδ T-cells, recovery of paired γδ TCRs, and generation of engineered TCR-T cells with broad cancer reactivity, including across tumour types and independently of β2M expression.

## Discussion

The absence of suitable tumour-specific TCRs remains a central bottleneck in TCR-based immunotherapy. While the clinical success of TIL therapy and immune checkpoint blockade demonstrates that TCRs capable of clearing metastatic cancers exist in some patients, there has been a persistent practical difficulty in finding these receptors, validating their function, and extracting their sequences rapidly and cleanly enough to drive therapy development. Conventional discovery workflows often rely on prior antigen knowledge, extended culture and cloning, or ICS approaches that compromise cell viability and therefore constrain downstream applications. These challenges are particularly acute when working with scarce, highly polyclonal clinical material, or when the most informative T-cell populations are rare, as is often the case for TIL products derived from patients with exceptional clinical responses. Here, we describe the T107 assay, a short co-culture and flow cytometry-based approach that identifies responding T-cells through simultaneous surface mobilization of CD107a and surface retention of TNF. Unlike cytokine-capture approaches such as IFNγ secretion assays, the T107 assay identifies responding T-cells through direct detection of TNF retention and CD107a mobilization during short co-culture, avoiding the need for a secretion-capture phase and reducing the risk of cytokine diffusion between neighbouring cells. This strategy enables recovery of live cancer-reactive T-cells without *a priori* knowledge of antigen specificity. In practice, this combination provides a robust functional readout that is directly compatible with downstream live-cell sorting, molecular profiling, and culture, rather than forcing an early choice between functional assessment and recoverability. In TILs from patient MM909.24, the assay produced a clear and discrete responding population, reached a plateau rapidly, and yielded a brighter TNF^+^ signal than matched ICS conditions, facilitating confident gating and sorting. Importantly for real-world workflows, surface TNF and CD107a staining remained stable for hours on ice without fixation, a practical consideration that often determines whether sorted cells and sequencing libraries succeed in shared cytometry facilities.

A key strength of the T107 assay is its breadth across T-cell lineages and phenotypes. Using clinical TIL products, we detected and characterized cancer-reactive αβ and γδ TCR^+^ T-cell populations spanning CD8 and CD4 co-receptor phenotypes, with patterns that were biologically consistent with tumour target properties such as MHC class II expression. These differences in tumour-reactive T-cell composition between patients likely reflect variation in tumour antigen presentation and microenvironmental context, including HLA class I and class II expression, as well as the potential contribution of HLA-independent recognition mechanisms such as those mediated by γδ T-cells. In addition, factors such as clonal expansion and *in vitro* culture may further influence the observed distribution of T-cell subsets. This is particularly relevant given that many existing discovery pipelines are implicitly biased towards HLA class I-restricted CD8^+^ αβ TCR^+^ T-cell populations. The antigen-agnostic functional capture step used in the T107 assay operates across T-cell subsets and increases the probability of recovering receptors with therapeutic relevance, including those that recognize non-canonical ligands or display unconventional restriction features. The value of sorting on function was especially clear in patient MM909.46, where sequencing of the T107-enriched fraction revealed dominant responding TCR chains that were present only at low frequency in bulk, unsorted TIL. This illustrates a recurring limitation of repertoire analysis: bulk sequencing is highly effective at describing *abundance*, but abundance does not equate to tumour reactivity. By enriching specifically for functional responders prior to sequencing, the T107 assay shifts the signal-to-noise ratio decisively towards receptors that actively recognize cancer, even when those clonotypes are rare in the starting population. In patient SAR26, the same T107 workflow supported parallel phenotyping, sequencing, and T-cell cloning from a single assay, yielding a functional αβ TCR^+^ CD8^+^ T-cell clone that killed autologous tumour cells and enabled rapid inference of the restricting element using engineered target knockouts. The ability to connect a functional readout directly to a cloned T-cell and then to a defined receptor sequence, without extended discovery cycles, substantially accelerates selection of promising TCRs from patient-derived material.

The T107 assay also enables isolation of orphan clonotypes for antigen discovery. From patient MM909.11, we recovered multiple cancer-reactive T-cell clones spanning private reactivity, shared melanoma-associated recognition, and recognition of antigens shared across distinct tumour types, all without prior knowledge of antigen identity. This progression, from tumour reactivity to restriction inference and ultimately to epitope identification, reflects how receptor discovery is performed in practice when antigen specificity is unknown. Importantly, the T107 assay facilitates identification of anticancer T-cells that extend beyond conventional peptide-HLA recognition paradigms. Using this assay, we have previously identified multipronged αβ TCRs such as MEL8, capable of recognizing multiple distinct cancer antigens via a single receptor [42]. MR1-restricted TCRs identified by this approach revealed a previously unappreciated mechanism of tumour recognition, exemplified by MC.27.759S [3, 41]. In the present study, we extend this framework to the discovery of cancer-reactive γδ TCRs capable of responding to multiple tumour types in an HLA-independent manner. A further strength of the approach is its compatibility with modern T-cell engineering strategies. From patient MM909.15, tumour-reactive γδ T-cells could be enriched, functionally interrogated against additional cancer types, and single-cell sorted for paired TCR recovery. These naturally occurring γδ TCRs were then functionally deployed by engineering αβ T-cells to express the recovered receptors, generating γδ TCR-T cells with broad cancer reactivity.

There are, however, limitations that should be acknowledged. A key consideration of the T107 assay is that detection depends on T-cell activation reaching a functional signalling threshold. As a result, very low-affinity interactions may not generate sufficient TNF production or CD107a mobilization to be captured. While sensitivity could in principle be increased by enhancing antigen density or providing additional co-stimulation, we deliberately avoided such interventions to preserve physiologically relevant tumour recognition. This design therefore prioritizes identification of T-cells capable of robust functional responses, accepting that very weak interactions may be underrepresented.

CD107a and TNF report degranulation and activation respectively, and therefore strongly enrich for functional effector responses, but may under-represent tumour-reactive populations that signal predominantly through alternative cytokines, exhibit slower kinetics, or adopt non-degranulating programmes. As with any activation-based sorting strategy, purity depends on gating strategy and the biology of bystander activation, and orthogonal validation remains essential, particularly when making inferences about dominant clonotypes. In addition, the assay relies on co-culture with relevant target cells, which is advantageous when autologous tumour lines are available but can be limiting when tumour material is scarce or cannot be propagated. Use of non-autologous targets carries the risk of identifying alloreactive rather than tumour-specific responses. In addition, while the T107 assay is antigen-agnostic and applicable across tumour types, its performance depends on the availability and functional quality of TILs. Although we demonstrate applicability beyond melanoma, including in sarcoma-derived TILs, TIL yield and reactivity can vary substantially between cancers, which may influence assay performance in some settings.

The practical relevance of TCR discovery is underscored by the recent regulatory approval of TCR-based medicines. TCR-engineered T-cell therapies developed by Adaptimmune [49] and TCR bispecific molecules developed by Immunocore [50] demonstrate that naturally occurring TCRs can be translated into effective cancer therapies. However, the limited number of clinically deployed receptors highlights how difficult it remains to identify suitable TCRs with the required specificity, potency, and safety. Improved methods for rapid, antigen-agnostic procurement of tumour-reactive TCRs therefore remain a key unmet need. Overall, the T107 assay addresses a practical and conceptual challenge in receptor discovery: how to identify and recover viable tumour-reactive T-cells rapidly, without knowing the antigen in advance, and in a form that directly supports sequencing, cloning, antigen discovery, and cell engineering. By converting a short functional interaction with tumour into a sortable live-cell phenotype, the assay reduces reliance on labour-intensive workflows and broadens access to clinically relevant αβ and γδ TCRs. We have already used this approach to uncover new mechanisms by which TCRs can recognize malignant cells, generating substantial interest in the field [3, 41, 42], and anticipate that wider application of the T107 assay will continue to reveal additional, currently unrecognized routes for targeting cancer with T-cell-based immunotherapies.

## Supplementary Material

ltag009_Supplementary_Data

## Data Availability

All data generated or analysed during this study are included in this article and its supplementary information files. Patient-level data are restricted because of ethical and privacy considerations. Further information may be made available from the corresponding author upon reasonable request and subject to appropriate approvals.

## Permission to reproduce

The authors confirm that all figures, tables and data included in this manuscript are original or reproduced with appropriate permission. No third-party material requiring additional permission has been included, unless otherwise stated.

## Clinical trial registration

Clinical material used in this study was obtained from patients enrolled in studies conducted at the Centre for Cancer Immune Therapy, Herlev Hospital, Copenhagen University Hospital, Denmark, including the clinical trial ‘T cell Based Immunotherapy for Melanoma’ registered at ClinicalTrials.gov (NCT00937625).
