# Supplementary material for: Optimized procurement of cancer-reactive T-cell receptors from clinical samples
Source: Immunother Adv. 2026 Jun 22;6(1):ltag009. doi: 10.1093/immadv/ltag009 (PMC13487637; doi:10.1093/immadv/ltag009)
Supplement: Supplementary file 1 [file ltag009_supplementary_data.docx]

# Optimised Procurement of Cancer-Reactive T-cell Receptors from Clinical Samples

# Supplementary Material

Phenotyping

TCR sequencing

TNF

**Supplementary Figure** 1: Schematic overview of the T107 assay for studies requiring recovery of viable cancer-reactive T-cells. **A**. T-cells are co-incubated with cancer cells in the presence of the TNF-processing inhibitor TAPI-0 and fluorescently-conjugated antibodies directed against TNF and CD107a. **B**. TAPI-0 inhibits the metalloprotease TNF-alpha converting enzyme (TACE), which normally cleaves membrane-bound TNF, thereby allowing surface retention and antibody detection of TNF on activated T-cells. **C**. CD107a becomes transiently exposed at the cell surface following fusion of cytolytic granules with the plasma membrane, enabling detection of degranulating T-cells by surface antibody staining. **D**. Following co-incubation with cancer cells, T-cells can be stained for additional surface markers to enable phenotypic analysis and sorting by flow cytometry. Viable cells can be recovered, as detection of TNF and CD107a does not require fixation or intracellular staining.

cell

Granzyme and Perforin

CD107a

CD107a

Ab

T-cell

Cell sorting

Acquisition

CD107a

Membrane bound TNF

Soluble TNF

TACE TAPI-0

TNF Ab

Cancer cell

T-cell

**A** T107 assay	**D** Flow cytometry	**E** Outcome

**B** TAPI assay + **C** CD107a assay

Lysosome

Cancer

T-cell culture

**E**. The T107 assay, combined with flow cytometry, enables phenotypic characterisation of cancer-reactive T-cells and isolation of viable cells for downstream applications, including T-cell receptor analysis and *in vitro* culture.

# C

150K

100K

50K

0K

**Lymphocytes**

0K	100K	200K

FSC-A	FS

**Single cells**

0K	100K	200K

C-A

250K

200K

150K

100K

50K

0K

100

80

60

1968

2222

40

20

0

100

80

_60_	9802

4395

40

20

0

0	10^3^	10^5^

TNF PE-Vio770

SSC-A

FSC-H

**A**

Key:	T107	ICS

A 3 hours

**Viable T-cells**

**Functional analysis**

10^4^

10^3^

10^5^

10^4^

10^3^

0

0

0

ViViD

10^3^

10^5^

0

10^3^	10^4^

CD107a PE

10^5^

10^3^

Autologous cancer cells

TILs

alone

CD3 PerCP

TNF PE-Vio770

4 hours
5 hours	6 hours

10^3^

% maximum

 CD107a PE

CD3/CD28

DynaBeads

TNF PE-Vio770

| 0 | 0.24% | 0.12% | 0.15% | 0.05% | 0.23% | 0.19% | 0.98% | 0.25% |
| --- | --- | --- | --- | --- | --- | --- | --- | --- |
| 10^5^ |  |  |  |  |  |  |  |  |
| 10^3^ |  |  |  |  |  |  |  |  |
| 0 | 33% | 30% | 39% | 40% | 38% | 39% | 41% | 40% |
| 10^5^ |  |  |  |  |  |  |  |  |
| 10^3^ |  |  |  |  |  |  |  |  |
| 0 | 97% | 97% | 97% | 98% | 98% | 99% | 98% | 98% |
| 0 | 10^3^ 10^4^ 0 | 10^3^ 10^4^ 0 | 10^3^ 10^4^ 0 | 10^3^ 10^4^ 0 | 10^3^ 10^4^ 0 | 10^3^ 10^4^ 0 | 10^3^ 10^4^ 0 | 10^3^ 10^4^ |

**Supplementary Figure 2**: **Gating strategy, incubation time optimisation and comparison of the T107 assay with intracellular cytokine staining. A**. Gating strategy for identification of cancer-reactive T-cells using the T107 assay. Lymphocytes were identified based on forward and side scatter properties, followed by exclusion of doublets and dead cells, gating on CD3-positive T-cells, and subsequent identification of reactive cells based on TNF and CD107a surface expression. **B**. TILs from melanoma patient MM909.24 were incubated with or without autologous melanoma cells using the T107 assay for the incubation times indicated. CD3/CD28 Dynabeads were used as a positive control. Comparable frequencies of CD107a-positive TNF-positive T-cells were observed across the incubation times tested, and 4 hours was adopted as the standard T107 assay duration. Performed in duplicate. **C**. Mean fluorescence intensity (MFI) of TNF and CD107a staining measured following the T107 assay (4-hour incubation) and intracellular cytokine staining (ICS) using MM909.24 TILs incubated with autologous melanoma cells. MFIs illustrate differences in signal intensity between the two assays.

**A**	0 hours

**UNFIXED**

1 hour post staining

3 hours post staining

| 10^4^ |  | | | | | | | | | | |
| --- | --- | --- | --- | --- | --- | --- | --- | --- | --- | --- | --- |
| 10^3^ |  |  |  |  |  |  |  |  |  |  |  |
| 0 |  | 0.23% |  | 0.31% |  | 0.23% |  | 0.26% | 0.05% |  | 0.61% |
| 10^4^ |  |  |  |  |  |  |  |  |  |  |  |
| 10^3^ |  |  |  |  |  |  |  |  |  |  |  |
| 0 |  | 27% |  | 29% |  | 29% |  | 30% | 31% |  | 30% |
| 10^4^ |  |  |  |  |  |  |  |  |  |  |  |
| 10^3^ |  |  |  |  |  |  |  |  |  |  |  |
| 0 |  | 95% |  | 97% |  | 95% |  | 95% | 91% |  | 96% |
| 10^4^ |  |  |  |  |  |  |  |  |  |  |  |
| 10^3^ |  |  |  |  |  |  |  |  |  |  |  |
| 0 |  | 0.03% |  | 0.10% |  | 0.28% |  | 0.36% | 0.12% |  | 0.36% |
| 10^4^ |  |  |  |  |  |  |  |  |  |  |  |
| 10^3^ |  |  |  |  |  |  |  |  |  |  |  |
| 0 |  | 29% |  | 27% |  | 27% |  | 27% | 27% |  | 28% |
| 10^4^ |  |  |  |  |  |  |  |  |  |  |  |
| 10^3^ |  |  |  |  |  |  |  |  |  |  |  |
| 0 |  | 91% |  | 98% |  | 86% |  | 94% | 97% |  | 93% |
|  | 0 | 10^3^ | 0 | 10^3^ | 0 | 10^3^ | 0 | 10^3^ | 0 10^3^ | 0 | 10^3^ |

CD107a PE

**FIXED**

TNF PE-Vio770

**B**	MM909.24

melanoma

sarcoma

melanoma

100

80

60

40

20

0

100

80

60

40

20

0

0

10^3^	10^5^	0

10^5^	0	10^3^	10^5^

0	10^3^ 10^4^

0

10^3^ 10^4^

0

10^3^ 10^4^

HLA (Class I APC or Class II FITC)

Key:	No stain	Stain

HLA class I

SAR26

MM909.15

**Supplementary Figure 3: T107 assay antibody stability and HLA staining of cancer cell lines. A**. Stability of TNF and CD107a staining on unfixed cancer-reactive T-cells following the T107 assay. TILs from melanoma patient MM909.24 were incubated with or without autologous melanoma cells, or with CD3/CD28 Dynabeads, for 4 hours using the T107 assay. Following antibody staining, samples were either left unfixed or fixed and maintained on ice for 1 or 3 hours as indicated. TNF and CD107a staining remained stable under these conditions, supporting downstream sorting of viable cells. Performed in duplicate. **B**. Surface expression of HLA class I and HLA class II molecules on cancer cell lines used in this study, assessed by flow cytometry.

HLA class II

% maximum

###### A SAR26 sarcoma MM909.15 melanoma

Key:

WT	No Stain

Stain

KO	No Stain

Stain

HLA-A2 KO

β2M KO

β2M KO

MFI	MFI	MFI

0	10^3^

HLA APC

10^5^

19.6

10844

21.4

53.9

0	10^3^

β2M APC

10^5^

33.6

57397

25.3

52.3

0	10^3^	10^5^ β2M APC

29.3

20611

27.2

32.7

##### OCI-M2 AML

β2M KO

MFI 29.5

1467

26.9

73.2

0

10^3^ 10^4^ 10^5^

Key:

WT	No Stain

Stain

KO	No Stain

Stain

**Kasumi-6 AML**

##### MONO-MAC-1 AML

β2M KO

β2M APC

β2M KO

MFI 27.4

7892

28.4

35

0

10^3^ 10^4^ 10^5^

0	10^3^

10^4^ 10^5^

MFI 33.5

65550

30.3

40.6

# B

αβ TCR FITC

| 10^4^ | 100% 100% | MFI 29829 | MFI 9607 |
| --- | --- | --- | --- |
| 10^3^ |  |  |  |
|  |  | MFI 29119 | MFI 9116 |
| 0 |  |  |  |
|  | 0 10^3^ 10^4^ 10^5^  γδ TCR APC | 0 10^3^ 10^4^ 10^5^  CD8 APC Vio770 | 0 10^3^ 10^4^ 10^5^  CD4 BV510 |

**Supplementary Figure 4:** Validation of HLA-A*02:01 and beta-2-microglobulin knockout cancer cell lines and phenotypic characterisation of the SAR26.4 T-cell clone. **A**. Surface staining of HLA-A*02:01 and β2-microglobulin (β2M) expression on cancer cell lines used in this study. Wild-type and knockout (KO) cell lines are shown for sarcoma cells derived from patient SAR26 (HLA-A*02:01 KO and β2M KO), melanoma cells derived from patient MM909.15 (β2M KO), and acute myeloid leukaemia (AML) cell lines (β2M KO). Mean fluorescence intensity (MFI) values are displayed according to the key. **B**. T-cell receptor and co-receptor phenotypic analysis of the SAR26.4 T-cell clone, performed in duplicate.

**A**	Alone

10^3^	0.61%

17.4%

0.69%

10^2^

0

MEL624

1.9%

MM909.06

1.84%

MM909.14

0.67%

MM909.15

0.7%

MM909.22

10^3^	1.75%

MM909.24

0.4%

SK-MEL-28

1.32%

10^2^

0

0

10^3^	10^4^

0

10^3^	10^4^

0

10^3^	10^4^

CD107a PE

Autologous	FM-82

10^3^

10^2^

0

TNF PE Vio770

Key of HLA class I typing:	A*01:01	A*03:01	B*15:01	B*40:01	C*03:04

HLA A HLA B HLA C

**B**	100	**C**	40

Normalised reactivity to maximum (%)

% TNF/CD107a+

50	20

0

Autologous Non-autologous	^0^

Melanomas

**Supplementary Figure 5: Characterization of 𝛼βTCR^+^/CD8^+^ T-cell clone JWH-367 derived from metastatic melanoma patient MM909.11**. **A**. Reactivity of the JWH-367 T-cell clone assessed by the T107 assay following 4-hour co-culture with autologous melanoma cells and a panel of partially HLA-matched melanoma cell lines. HLA class I types of the autologous and non-autologous melanoma cell lines are indicated in the accompanying key. **B**. Quantitative comparison of JWH-367 reactivity towards autologous and non-autologous melanoma cell lines. Reactivity values are normalised to the maximum response. **C**. JWH-367 clone tested against wild-type and β2-microglobuln knockout autologous melanoma cells.

**A**	10^5^

0.77%

FM-6

67%

FM-74

79%

MEL624

74.8%

MM909.15

10^4^

10^3^

0

Alone

Autologous	FM-82	SK-MEL-28

10^5^

TNF PE Vio770

10^4^

10^3^

0

|  | 0.33% |  | 2.47% |  | 4.68% |  | 0.15% |
| --- | --- | --- | --- | --- | --- | --- | --- |
| 0 | 10^3^ 10^4^ | 0 | 10^3^ 10^4^ | 0 | 10^3^ 10^4^ | 0 | 10^3^ 10^4^ |

# B

TNF PE Vio770

10^4^

CD107a PE

Alone

0.30%

90.4%

0.64%

2.74%

MM909.24	AsPC-1	SiHa

10^3^

0

10^2^

10^3^	10^4^

10^2^

10^3^	10^4^

10^2^

10^3^	10^4^

10^2^

10^3^	10^4^

CD107a PE

Key of HLA class I typing:	A*01:01	A*03:01	B*15:01	B*40:01	C*03:04

HLA A HLA B HLA C

100

% TNF/CD107a+

**C**

50

0

MM909.24

**D**	4000

2000

melanoma

MIP-1β (pg/mL)

0

**Supplementary Figure 6: Characterization of 𝛼βTCR^+^/CD8^+^ T-cell clone AR-149 derived from metastatic melanoma patient MM909.11**. **A** Reactivity of the AR-149 T-cell clone assessed by the T107 assay following 4-hour co-culture with autologous melanoma cells and a panel of partially HLA-matched melanoma cell lines. HLA class I types of the autologous and non-autologous melanoma cell lines, as well as AsPC-1 pancreatic cancer and SiHa cervical cancer cell lines, are indicated in the accompanying key. **B**. Reactivity of the AR-149 T-cell clone assessed by the T107 assay following 4-hour co-culture with melanoma cell line MM909.24, AsPC-1 pancreatic cancer cells, and SiHa cervical cancer cells. HLA class I typing of target cells is indicated in the key. **C**. Reactivity of the AR-149 T-cell clone assessed by the T107 assay following 4-hour co-culture with wild-type MM909.24 melanoma cells or MLANA (Melan-A) knockout MM909.24 melanoma cells generated by CRISPR–Cas9. Data are shown for triplicate assay conditions, with error bars representing standard deviation. **D**. Antigen-specific activation of the AR-149 T-cell clone following overnight stimulation with HLA-A*03:04-restricted peptides SAYGEPRKL (derived from MAGE-A1) and LAMPFATPM (derived from NY-ESO-1) at 10^-5^ M. Supernatants were collected for measurement of MIP-1β by ELISA. Phytohaemagglutinin (PHA) was used as a positive control.

# A

10^5^

10^4^

10^3^

Alone

Autologous

MEL624	MM909.15

0

0.49%

94.6%

90.8%

96.5%

0 10^3^ 10^4^

10^5^

FM82

0 10^3^ 10^4^ 10^5^

0 10^3^

MM909.14

10^4^ 10^5^	0 10^3^ 10^4^ 10^5^

MM909.24

10^5^

10^4^

10^3^

0

4.24%

0 10^3^ 10^4^ 10^5^

0.7%

0 10^3^ 10^4^ 10^5^

1.24%

0 10^3^ 10^4^ 10^5^

CD107a PE

TNF PE Vio770

Key of HLA class I typing:	A*01:01	A*03:01	B*15:01	B*40:01	C*03:04

HLA A HLA B HLA C

# B

0.6

0.4

0.2

0.1

0

0.6

0.4

0.2

0.1

0

TAG peptide sequence: RLSNRLLR

Position 1

Position 2

Position 3

A C D E F G H I K L MN P Q R S T VWY	A C D E F G H I K L MN P Q R S T VWY	A C D E F G H I K L MN P Q R S T VWY

Position 4

Position 5

Position 6

A C D E F G H I K L MN P Q R S T VWY	A C D E F G H I K L MN P Q R S T VWY	A C D E F G H I K L MN P Q R S T VWY

Position 9

A C D E F G H I K L MN P Q R S T VWY	A C D E F G H I K L MN P Q R S T VWY

0.6

0.4

0.2

0.1

0

Position 7

A C D E F G H I K L MN P Q R S T VWY

Amino acid residue (single letter code)

Position 8

MIP-1β (ng/mL)

**Supplementary Figure 7: Characterization of 𝛼βTCR+ CD8^+^ T-cell clone GD-22 derived from metastatic melanoma patient MM909.11**. **A**. Reactivity of the GD-22 T-cell clone assessed by the T107 assay following 4-hour co-culture with autologous melanoma cells and a panel of partially HLA-matched melanoma cell lines. HLA class I types of the autologous and non-autologous melanoma cell lines are indicated in the accompanying key. **B**. Antigen discovery for the GD-22 T-cell clone using positional scanning combinatorial peptide library (PS-CPL) screening and interrogation of the CANTiGEN database. The TAG-derived peptide RLSNRLLLR was identified as the peptide recognised by GD-22 and is highlighted in blue.

**A**	MM909.15 TILs

Subset % of cancer reactive

10	100

75

% TNF^+^ CD107a^+^

5	50

25

0	0

of αβ TCR^+^

Key for melanoma cells:	WT	β2M KO

**B**	Jurkat cells

% of maximum

# C

100

100

80

60

40

20

0

MFI 4166

24258

0	10^4^	10^5^

𝛿1 FITC

% of maximum

80

60

40

20

0

Donor 9433	Donor 948U

0	10^4^	10^5^	0	10^4^	10^5^

MFI 3106

11392

MFI 2799

6144

𝛿1 FITC

**D**	TCR-T KI versus KI/KO

UT KI

KI/KO

MFI 875

7563

13849

0	10^4^	10^5^

𝛿1 FITC

Key for B-D: Untransduced (UT) TMA TCR

**Supplementary Figure 8: β2-microglobulin dependence of tumour-infiltrating lymphocytes from patient MM909.15 and expression of the 𝛾4𝛿1 TMA TCR in Jurkat cells and primary CD8^+^ T-cells. A**. Reactivity of TILs from melanoma patient MM909.15 assessed by the T107 assay following 4-hour co-culture with wild-type or β2-microglobulin knockout (β2M KO) autologous melanoma cells (left). Subset analysis of cancer-reactive T-cells identified in response to wild-type and β2M KO melanoma cells is shown on the right. **B**. Expression of the 𝛾4𝛿1 TMA T-cell receptor derived from patient MM909.15 in T-cell receptor-deficient Jurkat cells. **C**. Expression of the 𝛾4𝛿1 TMA T-cell receptor in primary CD8+ T-cells from two healthy donors. **D**. Expression of the 𝛾4𝛿1 1 TMA T-cell receptor in primary CD8^+^ T-cells from a healthy donor generated either by TCR knock-in (KI) alone or by TCR replacement (KI/KO), in which the endogenous TCRβ chain was deleted using CRISPR–Cas9.
